# Naturalizing Phenomenology: A *Must Have*?

**DOI:** 10.3389/fpsyg.2018.01933

**Published:** 2018-10-22

**Authors:** Liliana Albertazzi

**Affiliations:** Laboratory of Experimental Phenomenology, Department of Humanities, University of Trento, Trento, Italy

**Keywords:** consciousness, experimental phenomenology, first person account, naturalization, phenomenology

## Abstract

Quite a few cognitive scientists are working toward a naturalization of phenomenology. Looking more closely at the relevant literature, however, the ‘naturalizing phenomenology’ proposals show the presence of different conceptions, assumptions, and formalisms, further differentiated by different philosophical and/or scientific concerns. This paper shows that the original Husserlian stance is deeper, clearer and more advanced than most supposed contemporary improvements. The recent achievements of experimental phenomenology show how to ‘naturalize’ phenomenology without destroying the guiding assumptions of phenomenology. The requirements grounding the scientific explanation of subjective experience are discussed, such as the nature of the stimuli, their variables, and their manipulation by properly phenomenological methods.

## Introduction: Cool Phenomenology

In recent years declaring oneself a phenomenologist, or of a phenomenological persuasion, has become a rather fashionable attitude among philosophers and scientists of cognitive neuroscience. Apparently, it is a sort of *must have*, in order to refresh the dated and unpopular role of reductionism. Both philosophers and scientists pay increasing attention to the perspectival nature of perceiving (Husserl, 1925/1977; Green and Schellenberg, 2018), the question of veridicality of the percept (Vishwanath, 2005; Koenderink, 2010; Rogers, 2014; Albertazzi, 2015a; Hoffman et al., 2015; Mausfeld, 2015; Singer, 2015), the scope and limits of psychophysics for the analysis of mental facts (Hoffman, 2013), and the qualitative aspects of experience (Doerschner et al., 2010; Wijntes et al., 2012; Pont, 2013; Fleming, 2014). Generally speaking, however, experimental research is mainly conducted in a representationalist, formalist, and increasingly in an inferentialist Bayesian framework (Maloney and Wandell, 1986; Knill and Richards, 1996; Kersten et al., 2004; Brainard et al., 2006; Yuille and Kersten, 2006; Pellicano and Burr, 2012; Teufel et al., 2013), which is acquiring the status of an unquestioned theory of perception (criticism in Vishwanath, 2005; Albertazzi et al., 2010; Koenderink, 2014, 2016; Albertazzi, 2015a). The reason for that is the ontological roots of psychophysics and neuro-physiology, which are still tacitly placed in classical physics: the perceiver is supposed to represent a set of objective properties of the external physical environment, encoded and instantiated by the neurons. Eddington, however, observed how difficult it is to treat the physical world as purely symbolic, because we are unavoidably relapsing and mixing symbols with concepts taken from the world of consciousness with those taken from physics (Eddington, 1928, p. xv). Phenomena of consciousness are definitively out of place in Newtonian mechanics: although *directly* given in subjective experiences, they are phenomenal properties difficult to identify because of their unclear status. Different from primary or physical properties (spatiality, solidity, hardness, weight, shape, size, position, motion, defined in terms of metric cues), colors, sounds, tastes, odors, lighting, etc., as *subjectively* perceived, are the domain of secondary qualities, classically ascribed to consciousness, not to the physical universe (Galilei, 1623/1957, 274). Consequently, and because of the lack of adequate categories and theories of measurement, they have been long considered not susceptible to *objective* measures, which for centuries left the field open solely to philosophical inquiry. Subjective phenomena have been *indirectly* observed, and analyzed from an external, third-person account, in classical behavioral psychophysics and (neuro)physiology. A different perspective considers consciousness as hard-wired in the brain or as effect of local supervenience (Kim, 1984; Crick and Koch, 1990, 2003; Chalmers, 1995, 1996; Tononi and Koch, 2008), i.e., part of the physical matter, which allows a variety of mechanistic “explanations” (Bechtel, 2008; Craver and Derden, 2013; Oizumi et al., 2014; Miłkowski, 2016).

Today, raising the question of the nature, relevance, and role of appearances in our subjective experience immediately brings back to the fore the thorny issue of their measurement and modeling; not to mention the resurgence in science of the old dispute between qualitative and quantitative (Albertazzi, 2015a). It is a true challenge, indeed, for current (syntactical) algorithmic computation to model qualitative, subjective, relational (color assimilation, acoustic trill), ecologically meaningful (appealing), even connotative (melancholic lighting, tasteful look) properties that behave differently depending on the context in which they appear. Moreover, it seems almost impossible to find robust *measurement units* for phenomena perceived in awareness, because of intrinsically subjective biases and their fleeting status (Husserl, 1966/1991); something even more troublesome of the problem raised by Hochberg (1982) on the difficulty to extract optical invariants by a glance in the visual field. Indeed, there are limits and constraints on the duration and size of a stimulus pattern that is effective at any time (see also Peterson et al., 2007). Although the diverse conceptions propounded in cognitive neuroscience declare in principle that they focus on mental processes maintaining different viewpoints on mind and cognition, they still rely on behavioral data and in so doing are unable to address *subjective, immediate experience*. In cognitive science, one finds some theories of mind (computational, situated, enactive, symbolic, etc.), but one does not find a phenomenological theory of consciousness (Libet, 2004; Albertazzi, 2018). On the whole issue of the *facts* of phenomenology weighs on the old debate on the nature of “qualia,” a legacy of British empiricism and the analytic tradition. Qualia, in fact, are usually viewed as sense data and/or concepts (for example, “red”). As such, qualia suffer from Dennett’s criticism (Dennett, 1991) about being ineffable, inner, and private, hence not susceptible to experimental testing. A classic example of this apparent defeat of subjective experiences is provided by the philosophical conundrum of the incomparability of the “red” in my mind and in yours. On the contrary, a little phenomenological imagination and an exact experimental procedure suffice to dissolve what is essentially a sophistic argument: for example, to make subjects produce and objectify their own color mental contents (Albertazzi and Da Pos, 2016 and below). However, the supposed explanatory power of veridicalism (Pizlo et al., 2014; Pizlo, 2015) is far from being confuted or defeated.

Another example of this nebulous situation are the claims of a phenomenological attitude, such as the primacy of experience over physical stimuli (in line with Merleau-Ponty’s phenomenology, Merleau-Ponty, 1962, 1964), which are sometimes ascribed to Gibson’s “direct” perception (Gibson, 1950, 1979), overlooking the basic physical reference of ecological physics (optic array); or in a more recent proposal, already mentioned, the embodied approach to perception (O’Regan and Noë, 2001; Noë, 2004), which upholds a theory of perception *as* action while at the same time sustaining the idea of an extended mind.

## The Catchphrase

The motto *naturalizing phenomenology*! in scientific circles is an umbrella expression comprising a series of different claims, conceptions, and formalisms, further unbalanced by different philosophical and/or scientific concerns (for a broad introduction to the topic see Petitot et al., 1999, Ch. 1). Generally speaking, to naturalize is intended as to “integrate into an explanatory framework where every acceptable property is made continuous with the properties admitted by the natural sciences” (Petitot et al., 1999, 1–2). However, one is ironically reminded of Husserl’s statement in *Philosophy as a rigorous science* (Husserl, 1910-11/1965), “we are fighting against the naturalization of consciousness”!; or Brentano’s daring definition of “physical” phenomena (colors, sounds, landscapes, etc.) as the inner objects of consciousness (i.e., appearances) (Brentano, 1874/1995, 79–80, 2015; Albertazzi, 2013a,b, 2015b). Specifically, according to Husserl, any attempt to ground epistemology on psychology, or any positive science, is nonsense not only at the start but at any point along the way (Husserl, 1913/1982).

The growing community of scientists interested in phenomenology comprises supporters of both so-called internalist (Searle, 1983) and externalist (Davidson, 1980; Noë, 2004; Chella and Manzotti, 2007; Gallagher, 2008; Honderich, 2014; Clark, 2016) philosophical theories of mind/brain which try to fill the gap between the sciences of nature and the science of mind; as well a series of Gestalt reformers with expertise in computational neuroscience shifting from phenomenology to more quantitative analysis of gestalt type phenomena adopting Bayesian mathematical models (Epstein and Hatfield, 1994; Wagemans et al., 2012). For a different perspective, that identifies mind and object of experience, see Manzotti (2016, 2017a,b).

The phenomenologist community is neither exclusive nor uniform. It includes visual phenomenologists (Madary, 2017), cognitive linguists (Lakoff, 1987), cognitive neuroscientists (Ramachandran and Hubbard, 2005), statistical ecologists and roboticists (Brooks, 1991), cognitive scientists (Käufer and Chemero, 2015), mathematicians working on neuro-geometrical models (Petitot and Tondut, 1999; Citti and Sarti, 2014; Petitot, 2017), or attempting to model a science of qualities (Albertazzi and Louie, 2016). To orient oneself in a slippery and overlapping land of definitions and proposals is not easy. They range from naïve realist (Gibson, 1979; Chemero, 2011) to consciousness oriented (Hoffman, 2008) to immanent realism (Albertazzi, 2005); and proponents of the extended mind (Clark and Chalmers, 1998) explaining perceiving as a process of motor-sensory integrations (O’Regan and Noë, 2001; Noë and O’Regan, 2002). Then there is a wide set of embodied enactivists (a review in Ward et al., 2017), from those grounding in neurophysiology their negation of the representationalist viewpoint in perception, such as autopoietic enactivism (Varela et al., 1991; Varela, 1996; Thompson and Varela, 2001; Thompson, 2004, 2007; Thompson et al., 2005; Thompson and Zahavi, 2007), radical enactivism (Hutto and Myin, 2013), sensori-motor enactivism (Di Paolo et al., 2017), etc. (see Roberts, 2018). Finally, those highlighting the role played by social interactions in cognition (Ramstead et al., 2016; Gallagher, 2017), and those interpreting neuronal activities (the data) in terms of phenomenological categories such as intentionality (Rizzolatti et al., 2001; Gallese and Lakoff, 2005). In the last cases, to name but a few that have triggered lively discussion in cognitive science, understanding others’ mental states and actions is seen as a product of “simulation” implemented by a mirror-mechanism. In so doing, phenomenology is reduced to the inter-personal sharing of the same kind of neural and cognitive resources (see also Goldman, 2000). Given the complexity of the phenomenological theory, and considering the current proposals in cognitive neuroscience, one has to conclude that the boundaries between the current claims of a generalized “return to things in themselves” are rather blurred.

## Categorial Misunderstandings

Among the opponents of a representationalist viewpoint, and among the (internalist) proposals that have arisen from neuroscience, particularly interesting is the embodied constructivism propounded by Maturana and Varela (1980). In highlighting the shallowness and the fragmentation of a representationalist theory of reality, usually advocated by the proponents of symbolic internalism (Chomsky, 1968; Piattelli-Palmarini, 1980; Fodor, 1983, 2009; Mahon, 2015), this proposal presents a viewpoint apparently close to the main tenets of phenomenology, to whose classical views it refers [from the structures governing subjective time (Varela, 1999) to the idea of epoché]. In so doing, embodied constructivism paves the way for the study of consciousness relying on traditional research methodologies based on third-person account. This option endorses the assumptions of cognitive neuroscience concerning the relationship between information processing and brain activity. In this regard, Metzger, among others, was explicit in stating that, on studying the laws governing visual phenomena, one has to proceed without any glance at physics, chemistry, anatomy, and physiology; instead, one should proceed *from within*, i.e., from the immediate percept of which *one is conscious* (Metzger, 1936/2006, 197) in first person account. Neuro-phenomenology, considered from the same viewpoint, fails to achieve its goal of being a remedy for the awkward problem of consciousness (Varela, 1996; Varela and Shear, 1999). Thus, the current level of inquiry (neuro-physiology) in consciousness easily offers the side to criticism from the perspective of the explicit anti-naturalist program of Husserl’s phenomenology. Someone may interject, again and again, that one is working toward a “revision” of phenomenology from the viewpoint of current scientific achievements that as such were precluded to Husserl. This sounds a bit like begging the question, however, because the original phenomenological program did *not* require for its fulfillment the availability of either more quantitative data or more refined technological tools like those now available for brain scanning. Spontaneously emerging self-organizing processes in the brain do *not* explain what a Gestalt whole is from a *conscious* and *qualitative* viewpoint (be it a snowy landscape, colors in the open fields or on a canvas, or even a perceived atmosphere in moonlight). These aspects cannot be looked for or found in physical structures, but in the organization laws ruling consciousness’ processes as such. The current state of affairs in consciousness studies is nicely highlighted by Goldstein on observing that the stimulus, first in the environment and then on the receptors, creates electrical signals in the nervous system, which through *a miraculous and still not completely understood process* become transformed into subjective experiences (Goldstein, 2009, 24, emphasis mine). When brain scanning reveals – for example, the spontaneous organization of grouping at low level – it only testifies to the existence of a correlation between phenomenological experiences and brain activity: a *correlation*, not an *explanation* of qualitative appearances in the visual field *as* neural firing. The focus of current research in color, for example, is on how the nervous system determines which wavelengths are discriminated by the eye, not on how the nervous system, for example, creates subjective colorful experiences (Goldstein, 2009, 206). Indeed, *different observables* should be, but aren’t always recognized as the objects of the different sciences of psychophysics (behavioral response to physical stimuli), neurophysiology (neural correlates), and phenomenology (appearances).

All in all, and notwithstanding their differences due to the specific research field and methodologies, current proposals of embodied cognition, whether internalist or externalist, generally reduce the mind to the brain and/or to a psychophysical body. In so doing, they share a fundamental reductionist stance that uses classical physics or biology as primary ontological referents for the explanation of subjective phenomena. Embodied and enactive approaches can be powerful methodological tools in behavioral and neurophysiological investigation, but their ultimate reliance on sensorimotor contingencies for the construction of mental content inevitably gives them a naïve realist flavor (see Vishwanath, 2005). These theories lack a categorial distinction among the levels of reality (Poli, 2001, 2006), the different degrees of perceived reality (reality being a perceptual attribute in phenomenological experience, see Metzger, 1941/1963; Michotte, 1957; Mausfeld, 2013), and the different explanations of facts according to their specific complexity. Sharing a widespread terminological ambiguity, the theories presented as a cognitive neuroscience of phenomenology tackle the issue of mind and consciousness in quantitative terms, i.e., in terms of stimuli and the elaboration of information contained within the stimuli, occasionally still referring to the classical conception of Shannon and Weaver (1949/1998). In this regard, more recently a proposal for an “integration information theory” of consciousness has been presented (Tononi, 2004, 2008; Oizumi et al., 2014). It asserts the need for a theory preliminary to any implementation: in other words, to proceed top-down in this research field, i.e., from phenomenology rather than from neural mechanisms. In their study, these authors propose a list of properties, postulates and axioms of consciousness in terms of mechanisms and the composition of elementary mechanisms in higher-order ones, etc. From a phenomenological viewpoint, however, consciousness is not a set of states, the organization rule is not compositionality (there are no atoms, features, atomic individuals in consciousness), space and time are subjective, allowing widespread so-called illusions, temporal dislocations and reorganization of the stimuli at the qualitative level (Vicario and Zambianchi, 1998), etc. Also the proposal of an “active information” (Hiley and Pylkkänen, 2001), although in principle interesting from an ontological viewpoint (it is based on Bohm’s theory, see Hiley and Bohm, 1991) falls short again from a strict phenomenological viewpoint, because it focusses on brain correlates. In other words, if axioms for consciousness have to be pinpointed, one should start from the original Husserlian phenomenological analyses (temporality, meaning, intentionality, etc.), no matter how difficult they can be. The work to a large degree is already there.

The basic issue is thus how to construct a series of axioms for perceiver-dependent entities, whose grammar is currently not yet fully known, if not from an *indirect* viewpoint (i.e., from time to time by language or by psychophysical and/or neurophysiological units of measurement). In fact, we do not yet have a thorough geometry of subjective visual space and its elements (partial *descriptive* attempts in Arnheim, 1954, 1982; Massironi, 2002; Albertazzi, 2015c), nor a general theory of subjective space-time continua; we do not have a thorough theory of qualities (partial attempts in Rausch, 1966), i.e., of the “matter” of appearances, whose existence is still located at the physical and/or biological level. There is a body of literature very close to the original tenets of phenomenology that can be a preliminary reference framework, but a thorough theory and the methods for a phenomenological *science* are still in their infancy: to build such a science, for example, one cannot resolve to methods such as introspection or to folk narratives, the most common interpretation of intersubjectivity conveyed by linguistic and or social sharing (Hutto and Kirchhoff, 2015).

Thus, it might be reasonable to take a break, start again from given experience, and fix some points on what is meant by phenomenology properly. In what follows, I shall consider a few basic issues in phenomenology addressed in both philosophical and scientific practices.

## Phenomenology Proper

To define oneself “a phenomenologist,” one should at least be faithful to the *core* of the original and classical meaning of the term (here I leave aside the historical reconstruction of the definition in the phenomenological movement. See MacLeod, 1968; Spiegelberg, 1982; Zahavi, 2006; Albertazzi, 2013b, 2015b). Phenomenology, in pure Husserlian terms, is a *descriptive* theory of the essence of lived experience (Husserl, 1913/1982, I, §75), i.e., of the nature of perceptual and mental contents; or, in a friendlier characterization, phenomenology means a *description* of *direct* experience as naïve and full as possible (Koffka, 1935/1999, Ch. 3). The devil is in the details, because at stake is not the description of a direct perception of physical *stimuli* from the environment, as for example in the Gibsonian ecological theory of (direct) perception. Certainly, phenomenology alone is not sufficient to understand perception fully. The study of perception needs different sciences: on the one hand phenomenology, on the other, biological, neurophysiological and physical science. What is mandatory, however, is to avoid blurring them together. A science of appearances, rigorously putting into brackets the physical concept of *nature* (Husserl, 1913/1982, I, §76) requires a radical viewpoint, but still remaining a science, with methods commensurate to that specific domain (Kanizsa, 1991, 43–44).

Description, and empirical demonstration, are the classic phenomenological tools, which are not usually questioned by natural sciences. The issue, however, is whether phenomenology can also be experimental and find a place among the *exact* sciences (Boring, 1950, 360). Several representative exponents of the phenomenological research in perception show that it can (Katz, 1911; Michotte, 1950/1991, 1954/1963, 1962; Thinès, 1977). The issue needs clarification, however. The methods of Gestalt psychology have been both descriptive (Koffka, 1935/1999, Ch. 3), and demonstrative, for example by showing how among the plurivocity of the stimuli only one obeys the laws of organization (Kubovy and Pomerantz, 1981; Kanizsa and Minguzzi, 1984; Wagemans, 2015). As to the experimental methods, instead, some scholars adhering to Gestalt ascribe the *explanation* of subjective experiences to psychophysics and neuro-physiology, keeping the split between description and explanation in phenomenology (Spillmann, 2009), and paving the way for its supposed naturalization. However, the essential difference between experimentation in psychophysics and neurophysiology, on the one hand, and experimentation in phenomenology on the other, resides first of all in their *different observables*: the phenomena in the scene being described, manipulated, and verified in experimental phenomenology are appearances (colors, tones, lines, dots, squares, more complex forms, and scenes), their dependent and independent variables pertaining to the same level of analysis. For example, the order of colors on the basis of their similarities and dissimilarities, based on subjective visual inspection alone, evinces well-structured perceptual categories. This allows an *explanation* (in this case, the opponent theory) (Hering, 1920/1964) at the phenomenological level first, and successively the construction of a model such as the Natural Color System. The phenomenological observation of colors also makes it possible to enrich the definitions of hue, brightness and saturation by other definitions concerning the mode of appearance of colors, i.e., their behavior in the visual field (Mausfeld, 2003). For example, the phenomenal definition of *pronouncedness* [the accentuated characteristic that makes a color marked or prototypical: a red with high pronouncedness is, for example, 1080-R (NCS notation)]; or of *insistence* or forcefulness [the fact that a color appears as most vivid or brightest in the field (Katz, 1935)]. In so doing, phenomenology *confutes* the standard definition of color parameters, such as saturation based on stimuli (i.e., excitation purity), and it *explains* phenomenal characteristics not reducible to stimuli, such as the fact that colors with greater insistence always tend to stand out *before* other colors: since red is more insistent than blue, it appears to stand closer to the beholder (Da Pos and Albertazzi, 2010). Another aspect of colors *explained* by phenomenological analysis is their capacity to carry emotional information (Da Pos and Green-Armytage, 2007; Da Pos and Valenti, 2007). Consider for instance the cold–warm appearance of colors (Ou et al., 2004; Xin et al., 2004). These basic characteristics of color perception are *not* in the stimuli and do *not* need any validation from a neurophysiological viewpoint. The fact that evidence for the theory of opponent colors is provided by the discovery of opponent neural channels (Jameson and Hurvich, 1955; MacLeod and von der Twer, 2003) or that successively this anatomo-physiological substrate is shown to be unable to explain the phenomenological quality of opponent colors (Valberg, 2001; Kuehni, 2004; MacLeod, 2010), does *not* invalidate the phenomenological nature and behavior of colors. In Husserlian terms, the “eidetic” (i.e., structural) analysis, description, and explanation of the color behavior is valid as such. Any neuroscientific tool, such as brain scanning, as Metzger repetitively maintained (Metzger, 1936/2006, 19), is *not* an explanation of subjective experience, because it is given in third-person account by an external observer and according to the constraints of the method of observation (whatever the status or the development of the actual research tools may be). On the other hand, subjective evaluations given in first-person account, which characterize experimental phenomenology, do not *necessarily* need to be expressed by fully verbal descriptions of own lived experiences, successively manipulated and delineated in units of meaning, etc. (Giorgi, 2009). This method, in fact, among other things, implies conceptual and linguistic competence, delay in expressing and communicating one’s own lived experiences, re-framing the core meanings, etc. Similar problems arise in attempts to “explain” phenomenology as a branch or a part of sociological and/or anthropological research. For example, although studies conducted on sensory perception among cultures [see the works by the Concordia Sensory Research Team (CONSERT)^1^] are very interesting from an anthropological viewpoint, they do not cover the field of phenomenological research (see Albertazzi, 2015a).

As regards the adoption of linguistic tools, experimental phenomenology comprises, *inter alia*, the Osgood differential semantics (OD) (Osgood et al., 1957). OD *is* a phenomenological method characterized by the precise calibration and the choice of pairs of contraries to be experimentally tested, relatively to the specific phenomena under observation, be they color appearances, perceived lighting, transparency, or spatial, pictorial, and cross-modal dimensions, etc. In this case the choice implies specific natural language competence (what are analyzed, in fact, are not physical dimensions!), and *results* from the specific design of the experiment. In other words, the preliminary discussion about the proper characteristics to be tested and the consequent choice is neither naïve nor given for granted. Applied in this way, and focusing on characteristics of the specific phenomena, to a certain extent OD succeeds in avoiding the ambiguities potentially emerging from descriptive narratives. Some opacity remains, however, due to the different conceptualizations shaping different languages: consider the differences in the more or less reddish or bluish appearance of “purple” colors in different cultures, for example in Italian and English [purple (porpora) and violet (viola)] (Albertazzi and Da Pos, 2016).

Experimental phenomenology methods, however, do not rely only on linguistic tools, such as OD; they also rely on non-verbal test such as tasks of choice, association tasks (although not in the psychophysical protocols), and production tasks. None of these methods, however, implies short reaction (metric) times, assuming that the same observation conditions produce the same perceptions: what is tested in phenomenology is *not* the behavioral response to the stimuli. In experimental phenomenology, the choice of a method follows a preliminary and detailed description of the phenomena under observation, and sometimes requires *dedicated training*, as in the case of the observation of color appearances. I give some examples. A task choice in experimental phenomenology may be ordering colors on the basis of their similarities. The participants are asked to order a series of gray patches (such as pentagonal shapes or circles) ranging from white to black, randomly spread on a table or presented on a screen and produce the ordered series without being instructed on the ordering criteria. All people, in all countries, of all ages and languages produce the same sequence: once the order has been completed, any change would appear contrary to the nature itself of the (achromatic) color sequence. The same applies to chromatic patches that are ordered according to their hue similarity. Almost all observers soon realize that the sequence is closed, in the sense that the head and the tail of the obtained line of colors are similar to each other and, therefore, must also be closer to each other in space. The procedure is so simple that it is universally used to differentiate defective from normal color viewers (Farnsworth, 1943 test). This task requires a unidimensional order, and for this reason observers do not encounter particular difficulties in reaching the final solution.

Another example of phenomenological methods concerns the conditions of appearances of transparent objects (3D objects, 2D surfaces, media). On inspection of the overlap zone where transparency is seen, the reducing color shows a specific characteristic (Ripamonti et al., 2017), i.e., it resembles both the underlying color surfaces. This condition has been simulated by the model of partitive mixtures (physical selective spectral filtering) (Metelli, 1974), on the hypothesis that the additive mixture of two colors produces a color similar to both. By remaining at the level of the phenomenological observation, instead, the condition of transparency is given by the fact that the reducing color must resemble both the colors, that of the transparent object and that of the object seen by transparency (Kanizsa, 1955). This condition predicts (according to the Natural Color System) that to see a transparent red object on a yellow background an orange surface has to be placed on the overlapping zone. Orange, in fact, is a color that *per se* resembles both red and yellow.

Another experimental phenomenological method is the sensory scale, first introduced by Da Pos and Pietto (2010) with the aim of studying perceived qualities of colors in terms of OD through a *sensorial* differential, avoiding verbal scales. This approach makes use of multisensory scales instead of the corresponding verbal expressions. Instead of asking the observer to rate on a bipolar scale how “cold” versus “warm” a musical clip is, the observer immerses his/her hands in a container of cold water (5° Celsius) and a container of warm water (40° Celsius), deciding which sensation is best associated between the two modalities (acoustics and touch) along a rating scale placed between the two sensory objects (for a follow up see Murari et al., 2017). More recently, experimental phenomenology methods have proved successful in the field of cross-modality (also with very complex scenes), such as paintings, poetry, and musical pieces, closer to our lived experiences than highly simplified ones (Albertazzi et al., 2015, 2016a,b). The phenomenological concept of scene (i.e., what we perceive), is often erroneously denoted with the term ‘stimulus.’ Unfortunately, the word ‘stimulus’ has different meanings in perception: on the one hand, it means the physiological stimulation of the sense receptors, on the other, the phenomenal content of a perception that can elicit a behavioral response. This confusion induces a theoretical confusion if we exchange stimuli with phenomenal events. In the above-mentioned experimental phenomenological studies, OD has usually been flanked by direct non-verbal associations.

Another method alternative to the non-phenomenological standard method was first introduced by Albertazzi and Da Pos (2016). The aim was on the one hand to identify what colors were associated with particular words in relation to a specific language (Italian) by objectifying them in color stimuli on the screen of a monitor; and on the other hand, to verify whether some words of that language denoted colors which were either particularly well defined or confused with others. Using a dedicated software, the subjects were asked to produce colors directly, instead of choosing among a number of colors presented on the screen. The procedure allowed the participants to produce those colors that corresponded, *in their minds*, to labels such as green, red, yellow, blue and to labels corresponding to perceptually mixed hues such as orange and lime. These methods, besides those rather well known of description and demonstration, show that experimental phenomenology can be conducted vigorously, i.e., that it is a science. *Phenomenology can be “naturalized*,*”* providing that one analyses and manipulates its observables through proper methodologies.

## On the Concept of Naturalizing

Some objections have been raised concerning the nature and value of experimental phenomenology. The first one concerns, as we saw, whether the Husserlian stance, “to fight against the naturalization of phenomenology,” allowing a descriptive and demonstrative application of the theory, precludes, however, its experimental development. In fact, it does not, as the examples mentioned above show. One has to understand what precisely Husserl had in mind in denying that subjective experiences could be subject to a natural science inquiry. One of the strongest arguments brought by Husserl “the mathematician” against the naturalization of phenomenology was based on his idea of the impossibility to mathematize subjective experiences as immanent and directly given in awareness (Husserl, 1925/1977, 1936/1970). Apparently, in fact, the *descriptive* procedure contrasts with the *axiomatic* procedure ruling the domain of mathematics, i.e., the deductive demonstration of theorems starting from axioms. Reasons for the intrinsic limits of the phenomenological inquiry were ascribed to the nature of the phenomena under observation (appearances, configurations of qualities in awareness), these being perceived in a flow of subjective time-space, considered impossible to “observe” from both the outside and the inside because of their fleetingness, and hence impossible to be objectified (Husserl, 1925/1977). This objection, however, is not justified, because experimental phenomenology shows that it is possible to objectify subjective experience and adequately measure it. Initially, the supposed “impossibility” of its measurement derived from the lack of an adequate measurement procedures. Since Stevens (1957) the quantification (measurement) of phenomenological magnitudes has been made available. Stevens modified psychophysical investigation using the method of estimating magnitude by observing that subjects are able to express the intensity of their sensations through answers like ‘more or less heavy,’ ‘more or less good’ (which are ‘qualitative’ subjective evaluations because they are not metric in the strict sense: see Wertheimer, 1938). In fact, sensations can be *measured* with several methods: for example, by assigning numbers to them in proportion to their intensity or by the bisection method (presenting two sensations indicating them as very weak and very strong and asking the subject to produce a third one midway between the two). The Stevens’ framework, however, has its problematic sides: for example, the psychophysical *explanations* of “perceived quality” (as, for example, ‘more or less heavy,’ ‘more or less good,’ or ‘more or less round’) are still made in terms of integration between “perceived” variables (good, round, heavy) and “just not perceptible” correlates (retina and brain); every single sensation is determined by several physical variables (Marks, 1974); and some properties (for example color) are not reducible or *explainable* solely in terms of physical properties (on the whole topic see Albertazzi, 2015a).

The second objection concerns the possibility of a mapping between psychophysical/neurophysiological results and phenomenological experiences. Behind this objection lay the various proposals of isomorphism hypothesized between physical stimuli and phenomenological subjective appearances, which is the same as saying that psychophysics and phenomenology are but *different viewpoints* on the *same* observables. Actually, the best proposal for this problem concerns the linking propositions (Teller, 1984) between the phenomenological and the physical world, i.e., “the claim that a particular mapping occurs, or a particular mapping principles applies, between perception and physiological states” (Teller and Pugh, 1983, 581).

The main point, however, is that the *observables* of psychophysics and those of phenomenology *are* different: for example, proximity is not identical to the metrical distance between points, because appearances are far from being metric cues; perceived illumination is not describable in terms of radiance or luminance, etc., because it is characterized by connotative dimensions completely unobtainable from the physical stimulus. The “stimuli” of the phenomenological science are appearances, the “objects as they appear” to awareness, not images *re*presenting physical objects. Moreover, appearances are mostly organized along a continuum of pairs of contraries (beautiful/ugly, soft/rough, warm/cold, etc.), often presenting a neutral point, two extremities and an intermediate state (iced/boiling, warm/cold, and neither warm nor cold), dimensions that must be analyzed *iuxta propria principia* (in the last case, for example, not trying to map subjective warmth of color or of perceived lighting on physical temperature). *Either phenomenology is a science in its own right, having its proper aims, observables, methods, and results, or it does not exis*t; although one has also to admit that for the time being it is easier to define what phenomenology *is not* than what phenomenology *is* from a scientific viewpoint. Husserl has been very clear on the nature, the claims, the target and the methods of the science of nature, as opposed to a science of subjective experiences, which explains his aversion to naturalization, i.e., the reduction of the phenomenological experience to physics. In defining the boundaries of the legitimacy of the natural science, Husserl was somewhat in agreement with Galileo, who relegated the analysis of secondary qualities (such as odors, tastes, sounds, and colors) to the domain of consciousness, for which analysis a mathematical language was to be excluded. The identification of the observables, the methods and the nature of a phenomenological science were and still are in their infancy.

## Conclusion

The issue of consciousness and its computability has become controversial in contemporary science. Although the scientists’ answers are diverse, the tendency to reduce mental facts to brain activities or neural correlates, by means of a very sharp Occam’s razor, prevails. I have shown that and how a systematic naturalization that is restricted to the phenomenological level is possible if avoids the temptation of neural or psychophysical reductionism. *Experimental phenomenology*, as an empirical science of phenomenology maintains the broader principles in its original analysis (Brentano and Husserl) but provides concrete examples that may not have been made explicit in previous expositions. *To naturalize phenomenology* (i.e., the science of consciousness), however, scientists are required to build protocols and to develop methods for subjective evaluations in first-person account of kinds of observables (qualities) that still lack a fully adequate categorization (Rausch, 1966; Albertazzi, 2015a). Experimental phenomenology shows the direction. The outcomes of recent experiments, in fact, are promising (Albertazzi et al., 2015, 2016a,b; Albertazzi and Da Pos, 2016), and they also show that complex scenes can be experimentally tested in a rigorous way. Whatever we may come to know about the brain (Marshall and Magoun, 1998), from the viewpoint of “the phenomenologist” (descriptive, demonstrative, and experimental) it does not offer any information on consciousness phenomena. Only after having performed a categorial analysis of the nature of the phenomena (on the path paved by Brentano and Husserl), and identifying the rules governing the deployment of conscious perceiving (the processor), and a proper methodology to adopt in experimental tests, can one address the issue of naturalization of consciousness. To be avoided is still the stimulus error, substituting the list of the characteristics of the physical stimulus for the description of direct unbiased experience, i.e., what one “knows” instead of what one “perceives” (Kanizsa, 1979). It is also worth noting that the impossibility of developing a mathematical model of subjective experience maintained by Husserl has to be understood according to the mathematics of his time, i.e., a mathematics *intrinsic to physics* (the Galileian concept of nature). For the time being we have fractions of a potential formalization of biology (Rosen, 1991; Louie, 2013), however, still very far for being comparable to the completeness of formalization in physics. New mathematical theories may offer possibilities previously not available for a formalization of consciousness, although for the time being this research field is still pretty sparse. For all these reasons, a phenomenological descriptive theory of experience in first person account is still the best option we have at disposal, also because it starts by showing that it is successfully susceptible to experimentation. Naturalization of phenomenology, however, needs a radical change of viewpoint on the concept of *nature* and of *consciousness*, and to consider mathematics not exclusively applicable to physics, but also to phenomenological science or phenomenology.

## Author Contributions

The author confirms being the sole contributor of this work and has approved it for publication.

## Conflict of Interest Statement

The author declares that the research was conducted in the absence of any commercial or financial relationships that could be construed as a potential conflict of interest. The reviewer DV declared a past co-authorship with the author.

## Footnotes

1 http://www.david-howes.com/senses/
